# Comparison of Different Tests to Assess Cardiorespiratory Capacity in Adult Male Football Players with Intellectual Disability

**DOI:** 10.3390/healthcare14050568

**Published:** 2026-02-25

**Authors:** Borja Suarez-Villadat, José Luis Maté-Muñoz, Juan Hernández-Lougedo, Ariel Villagra-Astudillo, Blanca Jiménez-Rojo, Fernando Jesús-Franco, Luis Maicas-Pérez, Pablo García-Fernández

**Affiliations:** 1 NÌKE Research Group, Faculty of Health Sciences, International University of La Rioja, 26006 Logroño, Spain; borja.suarez@urjc.es (B.S.-V.); jmate03@ucm.es (J.L.M.-M.); ariel.villagra@uam.es (A.V.-A.); blancajimenezrojo@gmail.com (B.J.-R.);; 2 Department of Physical Therapy, Occupational Therapy, Rehabilitation and Physical Medicine, Health Sciences Faculty, Universidad Rey Juan Carlos, 28922 Madrid, Spain; 3 Faculty of Nursing, Physiotherapy and Podiatry, Complutense University of Madrid, 28040 Madrid, Spain; 4 Physiotherapy and Health Research Group (FYSA), Faculty of Health Sciences-HM Hospitals, University of Camilo José Cela, Urb. Villafranca Del Castillo 49, 28692 Madrid, Spain; 5 Department of Physical Education, Sport and Human Motricity, Autónoma University of Madrid, 28049 Madrid, Spain; 6 Innovation and Health Department, Club Atlético de Madrid Foundation, 28022 Madrid, Spain

**Keywords:** Six-Minute Walk Test, intellectual disability, cardiorespiratory fitness, functional tests, adapted football, physical assessment

## Abstract

**Background:** Intellectual disability limits physical activity, affecting health and quality of life. Efficient tests to assess cardiorespiratory fitness in adapted football are essential. The Six-Minute Walk Test (6MWT) is a widely used benchmark test but can be logistically challenging. Although alternative tests such as the Sit-to-Stand Test (STST), Chester Step Test (CST), and Two-Minute Step Test in Place (2MST) have been validated in other populations, no study has examined their relationship with the 6MWT specifically in football players with intellectual disability, a population with unique physiological and cognitive characteristics. Therefore, this study reports the convergent validity between the 6MWT and these alternative field tests and describes the physiological responses to each test in football players with intellectual disability. **Methods**: Forty-two adult male football players with intellectual disability (mean age 27.1 ± 5.6 years) completed the 6MWT, STST, CST and 2MSPT. Physiological parameters, including heart rate, oxygen saturation (SpO_2_), and systolic and diastolic blood pressure, were recorded before and after each test. Pearson’s correlation coefficients were calculated to assess relationships among tests. **Results:** Strong, significant correlations were found between the 6MWT and the STST (r = 0.711), CST (r = 0.724), and 2MSPT (r = 0.683) (all *p* < 0.001). All tests induced expected changes in heart rate, blood pressure and oxygen saturation. **Conclusions:** The STST, CST and 2MSPT showed strong associations with the 6MWT and may serve as practical, safe and efficient complementary tools for field-based assessment of cardiorespiratory fitness in this population. These findings apply specifically to adult male football players with intellectual disability and should not be generalized to other populations with intellectual disability.

## 1. Introduction

Intellectual disability is characterized by a neurological dysfunction that results in limitations in the development of activities and/or participation [1]. The etiological factors of intellectual disability are diverse and can be classified as genetic, acquired (either congenital or developmental), environmental, and sociocultural [2,3]. The phenotypic and genetic heterogeneity of this disability accounts for approximately 1700 recognized conditions [4]. These limitations can directly affect participation in regular physical activities, which in turn negatively impacts the overall health and quality of life of individuals with intellectual disability [5], particularly in the context of adult male athletes participating in adapted football programs.

In the field of sports, particularly in disciplines like football, assessing the physical fitness of athletes with intellectual disability is essential—not only to enhance performance but also to safeguard their health and promote their inclusion in competitive and recreational activities [6,7,8].

Among the components of physical fitness, cardiorespiratory capacity holds a central role due to its strong association with cardiovascular risk, daily functional ability, and life expectancy [9]. In individuals with intellectual disability, this capacity is often reduced compared to the general population due to factors such as sedentary behavior, obesity, and structural or social barriers to sports participation [10,11]. For this reason, functional assessment using specific physical tests is especially relevant for this population, particularly for athletes engaged in structured programs such as LaLiga Genuine, a competition specifically designed for individuals with intellectual disability.

Traditionally, the Six-Minute Walk Test (6MWT) has been considered the gold standard for evaluating cardiorespiratory fitness in people with intellectual disability, due to its simplicity, safety, and validity across clinical and athletic settings [12,13]. However, administering this test may be limited in contexts where space, time, or athlete cooperation is constrained. For this reason, alternative tests such as the Chester Step Test (CST), the Two-Minute Step Test (2MSPT), and the Sit-to-Stand Test (STST) have been explored. These offer logistical and operational advantages by requiring less equipment, space, or execution time [14,15,16].

The appropriate selection of functional tests not only streamlines the evaluation process but also facilitates tailored training based on objective data. Specifically, for football players with intellectual disability, having reliable and practical tools to assess cardiorespiratory fitness can make a significant difference in training planning and injury prevention, as well as in enhancing athletic performance and overall quality of life [17].

Despite the widespread use of the 6MWT and the availability of alternative field-based assessments, evidence remains limited on how common functional tests compare within the same cohort of football players with intellectual disability, a population in which feasibility, comprehension, and safety considerations may influence test choice and interpretation. In particular, most previous work examining the CST, STST, or 2MSPT has been conducted in other clinical or community populations, leaving uncertainty about their convergent performance with the 6MWT and their physiological demands in sport contexts involving athletes with intellectual disability.

Therefore, the present study aims to examine the convergent validity of common functional field tests by assessing their associations with the 6MWT and by describing the physiological response profiles elicited by each test in football players with intellectual disability. This head-to-head comparison is intended to support the selection of accessible, valid, and adaptable assessment methods in sports and educational environments where individuals with intellectual disability are actively involved.

We hypothesized that performance in the STST, CST, and 2MSPT would show moderate-to-strong positive associations with 6MWT distance (convergent validity), and that the CST would elicit greater end-test cardiovascular responses than the other field tests.

## 2. Materials and Methods

### 2.1. Design and Participants

A convergent validity analysis was conducted to examine the relationship between different field-based functional tests used to assess cardiorespiratory fitness in football players with intellectual disability. All participants or their legal guardians signed an informed consent form prior to the start of the assessments. The study was approved by the Ethics Committee of the International University of La Rioja (approval number: PI:062/2023) and was conducted in accordance with the ethical principles outlined in the Declaration of Helsinki. This study was conducted in accordance with the STROBE (Strengthening the Reporting of Observational Studies in Epidemiology) guidelines for observational studies.

Participants were selected based on the following criteria: they had to present a clinical diagnosis of mild or moderate intellectual disability, be between 18 and 35 years of age, regularly attend football training sessions (at least twice a week), and demonstrate the ability to understand and follow simple instructions related to the testing procedures. Individuals with uncontrolled cardiovascular, respiratory, or neurological conditions; those with musculoskeletal injuries limiting physical activity; or those displaying behavioral issues that could compromise test safety were excluded. Lack of informed consent from the participant or their legal guardian also resulted in exclusion from the study.

Prior to the start of the study, data on age and anthropometric characteristics of participants were collected. The primary outcome was physical fitness, assessed through the following field-based tests: Six-Minute Walk Test (6MWT), One-Minute Sit-to-Stand Test (STST), Chester Step Test (CST), and Two-Minute Step Test in Place (2MSPT). All tests were performed at the Research Center for Sport and Health of the Atlético de Madrid Foundation. The test order was determined by prior randomization. Before the test battery, participants completed a brief standardized warm-up (low-intensity walking/marching and dynamic lower-limb mobility). For each test, the assessor provided a standardized explanation and demonstration; a short practice period was allowed when needed to ensure comprehension and safe execution in this population. Adequate rest periods were provided between tests (30 min), allowing oxygen saturation (SpO_2_) and heart rate (HR) to return to baseline values, with a minimum of 30 min between trials.

### 2.2. Safety Monitoring and Stopping Criteria

Participants were continuously observed by trained staff throughout each test. Prior to each assessment, participants were screened for acute symptoms, and baseline vital signs were recorded. Heart rate was monitored using a Polar H9 chest strap (Polar Electro Oy, Kempele, Finland) and oxygen saturation (SpO_2_) were monitored using a pulse oximeter PO-80 (Beurer GmbH, Ulm, Germany) during the tests and reassessed immediately after completion; blood pressure was measured using an OMRON M2 Essential (OMRON Healthcare Co., Ltd., Kyoto, Japan) before and immediately after each test. Any adverse events or symptoms (e.g., dizziness, dyspnea, headache, chest discomfort, loss of balance/fall, or participant distress) were documented. Tests were terminated if any of the following occurred: (1) inability to maintain the required pace/technique despite standardized cueing; (2) onset of concerning symptoms; (3) reaching ≥90% of predicted maximal heart rate (HRmax = 210 − [0.65 × age]); (4) clinically significant oxygen desaturation, defined as SpO_2_ < 90% or a decrease of ≥4 percentage points from baseline [18]; (5) blood pressure values meeting predefined safety thresholds (e.g., SBP ≥ 250 mmHg and/or DBP ≥ 115 mmHg); or (6) voluntary cessation at any time. All tests were conducted twice due to the learning effect described in some field tests [19]. The entire evaluation team had prior experience performing these assessments.

### 2.3. 6MWT

The test was conducted following the guidelines of the European Respiratory Society and the American Thoracic Society [20]. Before starting, participants rested seated for at least 10 min, during which pulse, oxygen saturation, and blood pressure were measured. Participants were instructed to walk as far as possible along a 30 m flat corridor marked with cones for six minutes. An evaluator, trained in the 6MWT protocol, walked alongside the participant, used standardized phrases, and informed them of the time elapsed each minute. Participants could rest if needed, though the stopwatch continued running. The number of laps and total distance walked were recorded. Results were expressed as total distance walked in meters.

### 2.4. CST

Prior to the first CST attempt, participants received standardized instructions and a demonstration of the stepping technique (foot placement, full step-up/step-down, and turning). A brief familiarization was performed at Level 1 to ensure understanding of the task and the cadence cueing. The CST cadence was guided by a metronome according to the standard protocol; adherence to cadence was monitored continuously and reinforced using standardized verbal cues and, when needed, simple visual cues to support comprehension. The stepping rate itself was not modified. The test was performed on a 30 cm step and progressed by levels up to a maximum of 10 min. The maximum duration was 10 min [21]. Heart rate, oxygen saturation (SpO_2_), and blood pressure were monitored before, during (at the end of each stage), and immediately after the test. The test was terminated when (1) the participant could not maintain the required pace; (2) symptoms such as dizziness, dyspnea, or headache appeared and were immediately reported; (3) 90% of the predicted maximal heart rate (HRmax = 210 − [0.65 × age]) was reached; (4) oxygen desaturation occurred, defined as SpO_2_ < 90% or a decrease ≥4% from baseline; or (5) the participant voluntarily decided to stop. Results were expressed as the total number of steps completed.

### 2.5. STST

The test recorded the number of times the participant could rise from and sit back down on a chair within 1 min. A standard armless chair with a seat height of 17 inches (43.2 cm), rubber-tipped legs, and positioned against a wall was used. Participants were seated upright, arms crossed over the chest, and feet shoulder-width apart and slightly behind the knees. Two practice repetitions were performed before the actual test. Participants were encouraged to perform as many repetitions as possible within one minute [22]. Heart rate, SpO_2_, and blood pressure were monitored before, during, and after the test. The test was stopped under the same criteria as in the CST. Results were expressed as the number of sit-to-stand repetitions completed in one minute.

### 2.6. 2MSPT

Participants marched in place as fast as possible for 2 min, raising their knees to a midpoint between the iliac crest and the patella. A marker was placed on the wall to indicate the required knee height. Participants were allowed to use one hand for balance. Only the number of times the right knee reached the target height was counted. If the participant failed to reach the target, they were instructed to slow down to continue the test without interruption [23]. The test was terminated using the same criteria as the previous tests. Results were expressed as the number of steps completed.

### 2.7. Sample Size Calculation

The sample size was calculated using G*Power (v.3.1.9.7; [Heinrich-Heine-Universität Düsseldorf, Germany]) [24]. Given that the aim of the study was to analyze the association between the Six-Minute Walk Test (6MWT) and various alternative functional tests (STST, CST, 2MSPT), an a priori power analysis was performed for a bivariate Pearson correlation model, assuming a two-tailed test. The analysis was designed to detect a correlation of at least r = 0.50, using a significance level of α = 0.05 and a statistical power of 1 − β = 0.80. Under these parameters, the required sample size was 39 participants. Additionally, a potential dropout rate of 5% was considered, and thus a total of 42 participants was planned for recruitment. This sample size allows for the detection of moderate correlations between the different functional tests and the 6MWT, ensuring the statistical robustness and validity of the results obtained.

### 2.8. Statistical Analysis

The IBM SPSS Statistics (v.29.0; IBM Corp., Armonk, NY, USA) was used for all statistical analyses. The Shapiro–Wilk test was applied to assess normality. Depending on data distribution, parametric or non-parametric tests were used. Differences in physiological variables (heart rate, oxygen saturation, systolic and diastolic blood pressure) across tests were examined using analysis of variance (ANOVA) with Tukey’s post hoc test or the Kruskal–Wallis test, as appropriate; when omnibus tests were significant, pairwise post hoc comparisons were performed to identify which tests differed.

To evaluate convergent validity, associations between the 6MWT and the other functional tests were assessed using Pearson’s correlation coefficient (r), and 95% confidence intervals (95% CI) were calculated using Fisher’s z transformation. To account for multiple testing across the three prespecified correlations (6MWT vs. STST, CST, and 2MSPT), *p*-values were adjusted using the Benjamini–Hochberg false discovery rate (FDR) procedure.

In addition, multivariable linear regression models were fitted with 6MWT distance as the dependent variable and each alternative test as the independent variable, adjusting for age, body mass index (BMI), and the position of the 6MWT within the randomized testing sequence (1–4).

Data were presented as mean ± standard deviation (SD), 95% CI, minimum and maximum. A *p*-value < 0.05 was considered statistically significant.

## 3. Results

The results of the study are presented below.

Descriptive data are provided for the anthropometric variables (age, weight, height, and BMI) (Table 1) as well as the performance outcomes of the participants in the different functional tests administered (Table 2).

Final values of the physiological variables recorded before and after each of the functional tests, along with the statistical significance values obtained through comparative analysis between the tests.

Regarding baseline physiological variables, no statistically significant differences were observed among the four functional tests analyzed. Resting values for oxygen saturation, heart rate, and both systolic and diastolic blood pressure were comparable across the different assessments. These results indicate adequate homogeneity in the participants’ initial physiological status, thereby supporting the validity of subsequent comparisons related to exercise responses (Table 3).

Regarding the physiological variables recorded after completion of the functional tests, statistically significant differences were observed across assessments. Pairwise post hoc comparisons showed that the CST differed significantly from the other tests in final heart rate, systolic blood pressure, and diastolic blood pressure. For oxygen saturation, the CST differed from the 6MWT and the STST, but not from the 2MSPT. These findings reflect distinct physiological demands associated with each test, with the Chester Step Test eliciting the greatest cardiovascular response. Overall, the results indicate that, while all tests were safe, they produced different activation profiles, which may be useful when selecting the most appropriate test according to the specific objectives of the assessment (Table 4).

The Pearson correlation analysis revealed statistically significant associations between the Six-Minute Walk Test (6MWT) and the three alternative functional tests analyzed (STST, CST, and 2MSPT). These positive correlations indicate that all the tests assessed share a considerable degree of association with the cardiorespiratory capacity estimated by the 6MWT. All correlations remained statistically significant after Benjamini–Hochberg FDR correction (Table 5).

Multivariable linear regression models were fitted with 6MWT distance as the dependent variable. Three independent models were run, each including the performance in one functional test (STST, CST, or 2MSPT) as the main predictor, while age, BMI, and the position of the 6MWT within the randomized testing sequence (order 1–4) were included as covariates to control for potential confounding.

Overall, all three models showed positive and statistically significant associations between each test and 6MWT distance, providing evidence of convergent validity. Comparatively, STST showed the strongest association and the greatest explanatory power for 6MWT performance, followed by 2MSPT, whereas CST remained significantly associated but with a more modest relationship. These findings suggest that, within this test battery and population, STST and 2MSPT capture the construct assessed by the 6MWT more closely, while CST, although convergent, provides a weaker signal (Table 6).

**Figure 1:**
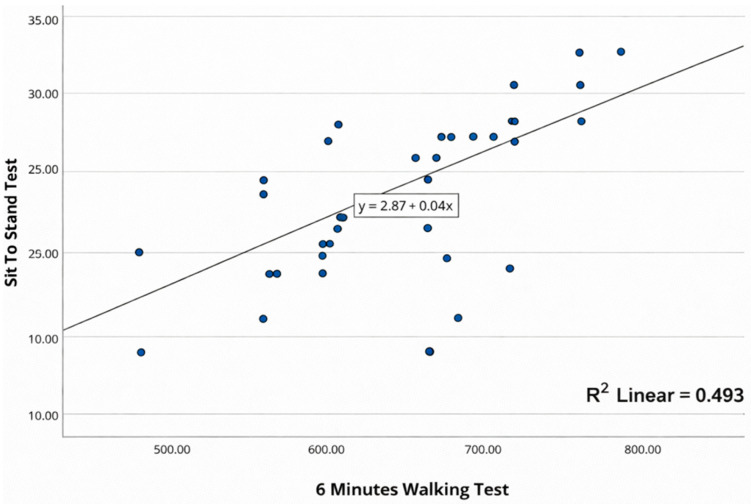
Scatter plot showing the correlation between 6MWT and STST.

**Figure 2:**
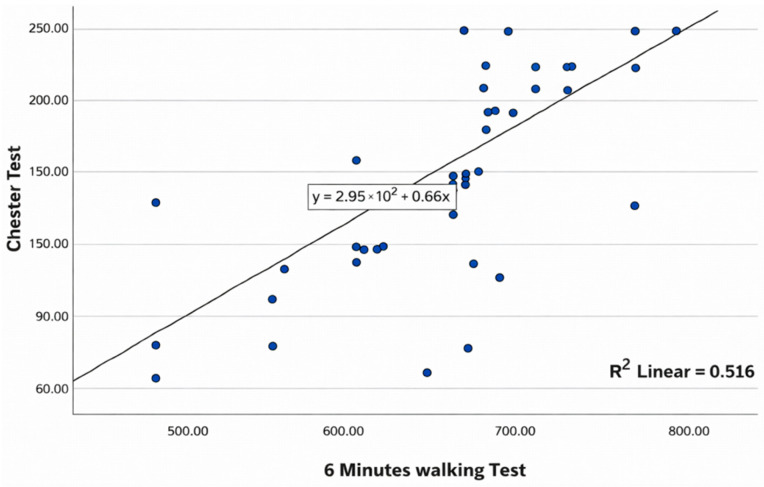
Scatter plot showing the correlation between 6MWT and CST.

**Figure 3:**
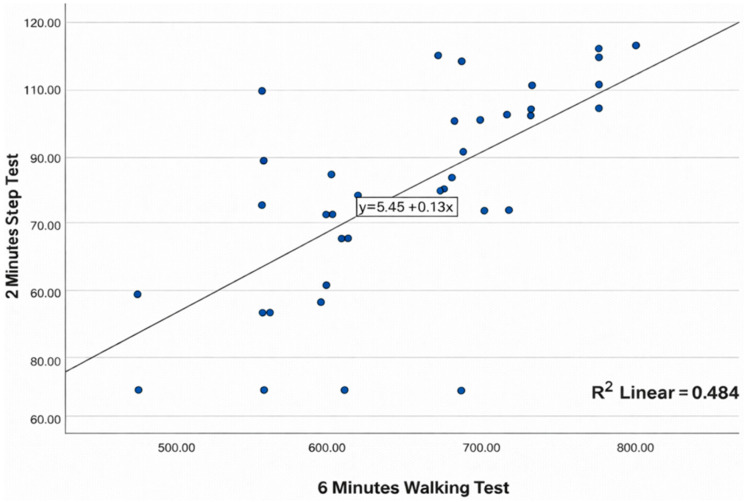
Scatter plot showing the correlation between 6MWT and 2MSPT.

The scatter plots illustrate the association between the Six-Minute Walk Test (6MWT) and each of the alternative functional tests are presented in Figure 1, Figure 2 and Figure 3.

The scatterplot shows the relationship between the 6MWT (*x*-axis) and the STST (*y*-axis). A positive linear trend is observed, such that better performance on the 6MWT is associated with higher STST values. The superimposed line corresponds to the linear regression fit, and the dispersion of the points suggests a moderate association between both tests (Figure 1).

Figure 2 shows the association between the distance covered in the 6MWT and performance on the CST. Overall, the data points follow an upward pattern, indicating that participants with better 6MWT results also tend to achieve higher values on the CST. The fitted line summarizes the linear trend, and the variability around it reflects a moderate relationship between both functional measures.

Figure 3 depicts the relationship between performance on the 6MWT and the 2MST. The results suggest that as the distance covered in the 6MWT increases, 2MST values also tend to rise, showing an upward linear trend. The fitted line summarizes this relationship, and the scatter around it indicates a moderate association between these two functional capacity tests.

## 4. Discussion

This study provides evidence of convergent validity, showing that performance in the STST, CST, and 2MSPT is associated with 6MWT distance in individuals with intellectual disability. These correlations, with Pearson coefficients of r = 0.711 (STST), r = 0.724 (CST), and r = 0.683 (2MSPT), support the validity of these tests as complementary or alternative tools for the 6MWT in contexts where its implementation may be limited. It is important to note that the present sample consisted exclusively of adult male football players from a single adapted football program. Therefore, these findings should not be generalized to women, younger individuals, or people with intellectual disability who do not participate in structured football training.

Previous literature has established the 6MWT as a valid and reliable test for individuals with intellectual disability [12,13]. However, its application may be hindered by logistical constraints such as the need for a large space, extended time, and sustained cooperation from the participant. In this regard, the present study provides empirical evidence supporting the use of shorter and more logistically feasible tests, which is particularly relevant in adapted sports or institutional settings with limited resources.

The CST showed the highest correlation with the 6MWT. This finding aligns with the work of Sykes and Roberts [14], who highlighted the CST’s utility as a progressive submaximal test for estimating aerobic capacity. In our study, the CST also produced the highest physiological responses, with significant increases in final heart rate (HR) (153.4 ± 21.3 bpm) and systolic blood pressure (SBP) (137.6 ± 18.1 mmHg), suggesting a greater cardiovascular demand. This response is consistent with studies such as Wiles et al. [25], which link exercise intensity with proportional increases in hemodynamic variables. However, its application requires greater motor coordination and technical supervision, which may limit its use among individuals with more severe cognitive or motor impairments.

The STST, in contrast, showed a strong correlation with the 6MWT and stands out for its simplicity, brevity, and minimal equipment requirements. Kronberger et al. [26] and Segura-Ortí et al. [27] have validated this test as a reliable indicator of lower-limb muscular endurance and general functional capacity. In the present study, the STST induced moderate physiological responses, with a final HR of 82.7 ± 17.6 bpm and SBP of 123.6 ± 13.4 mmHg, indicating a sufficient cardiovascular load without compromising participant safety. Its ease of administration makes it an ideal tool for routine assessments in sports or clinical settings.

The 2MSPT also showed a significant correlation with the 6MWT. While this test has been validated in older adults and patients with chronic conditions [16,28], its application in individuals with intellectual disability remains relatively novel. In our study, the 2MSPT elicited a final HR of 85.0 ± 19.1 bpm and SBP of 129.0 ± 12.2 mmHg, values that reflect adequate cardiovascular activation. Its short duration and ease of execution make it particularly useful for repeated assessments or for populations with low exercise tolerance.

From a physiological perspective, all tests produced expected increases in HR, SBP, and diastolic blood pressure (DBP), confirming their capacity to induce a valid cardiovascular response. The significant differences observed between tests in these variables (*p* < 0.001) reinforce the idea that each imposes a distinct load, which can be leveraged to select the most appropriate test depending on the evaluation objective. Variations in oxygen saturation were mild and not clinically significant, suggesting that all tests are safe for this population when applied under proper supervision.

The need for accessible and efficient assessment methods is particularly relevant in the field of adapted sports. Studies such as those by Gallotta et al. [6] and Žalienė et al. [7] have shown that participation in inclusive sports programs improves physical fitness and overall health in individuals with intellectual disability. However, the lack of adapted assessment tools may hinder the planning and monitoring of such programs. In this context, the findings of the present study offer viable alternatives that can be implemented without compromising the validity of the results.

Moreover, the literature emphasizes the importance of adapting tests to the cognitive and physical capacities of participants. Oppewal et al. [11] and de Leeuw et al. [29] highlight that individuals with intellectual disability often present lower fitness levels and higher cardiovascular risk, making the selection of safe, brief, and easy-to-understand tests even more critical. In this regard, the STST and 2MSPT emerge as particularly suitable options, as they require simple instructions and can be successfully completed by most participants.

Although the present study used a cross-sectional design and therefore cannot evaluate longitudinal sensitivity or training-induced changes, future research should investigate whether the CST, STST and 2MST are capable of detecting improvements over time following structured exercise interventions. Previous studies in individuals with intellectual disability have shown that structured training programs can lead to meaningful improvements in cardiorespiratory fitness [17,30], but the longitudinal responsiveness of the CST, STST and 2MST has not yet been examined in football players with intellectual disability. Establishing this responsiveness would be an important step toward integrating these assessments into monitoring and individualized training programs in adapted sport.

The findings of this study also offer practical guidance for coaches, physical educators, and therapists working with athletes with intellectual disability. The validation of short and accessible functional tests such as the STST, CST, and 2MSPT provides professionals with reliable tools to assess cardiorespiratory fitness in field settings. These tests require minimal equipment and time, making them ideal for routine evaluations during training sessions or physical education programs. Coaches can use these assessments to monitor progress, individualize training loads, and detect early signs of fatigue. Therapists may also incorporate these tests into rehabilitation protocols to evaluate functional improvements and cardiovascular responses in a safe and controlled manner.

### 4.1. Limitations

This study has several limitations. First, correlations quantify association but not agreement; thus, our findings support convergent validity with the 6MWT but do not establish criterion validity, agreement, or interchangeability between tests. Second, the sample included only male football players with intellectual disability, limiting generalizability to females and to individuals with different functional levels or contexts. Third, the modest sample size (n = 42) may reduce statistical power and the robustness of the observed associations. Fourth, no direct cardiopulmonary measures (e.g., Maximal Oxygen Uptake (VO_2_max)) were collected to provide a physiological criterion. Finally, the cross-sectional design precludes causal inference and assessment of change over time; therefore, longitudinal studies are warranted to evaluate maturational effects and responsiveness to training.

### 4.2. Future Research

Future studies should aim to replicate these findings in larger and more diverse populations, including female athletes and individuals with varying degrees of intellectual disability. Longitudinal research is needed to determine the sensitivity of the STST, CST, and 2MSPT to training-induced changes in cardiorespiratory fitness. Furthermore, integrating direct physiological measurements such as VO_2_max or lactate thresholds would enhance the validity of these field-based assessments. It would also be valuable to explore the feasibility and acceptability of these tests in real-world environments such as schools, community centers, and sports clubs. Agreement analyses (Bland–Altman plots/limits of agreement) and reliability/consistency metrics (ICC), ideally complemented by equivalence testing, are warranted in future work. Finally, future research should examine the relationship between performance on these functional tests and other health indicators, including cognitive function, quality of life, and physical activity participation.

### 4.3. Practical Applications

In clinical, sport, and educational settings, selecting a functional field test should balance feasibility, participant comprehension, available equipment/time, and the physiological load imposed by the assessment. Given their convergent associations with the 6MWT, the STST and 2MSPT may be preferred as rapid, low-resource options when space or logistics limit walking-based testing, whereas the CST may be useful when a higher cardiovascular load is desired and appropriate monitoring is available. These practical implications should be interpreted cautiously because no VO_2_-based measures were collected and the sample comprised only male football players with intellectual disability, which may limit generalizability to females and other populations or contexts.

## 5. Conclusions

This study confirms that the Sit-to-Stand Test (STST), Chester Step Test (CST), and Two-Minute Step Test (2MSPT) are valid alternatives to the Six-Minute Walk Test (6MWT) for assessing cardiorespiratory fitness in football players with intellectual disability, as evidenced by their positive and statistically significant correlations.

All tests demonstrated physiological safety, with heart rate, blood pressure, and oxygen saturation values remaining within clinically acceptable ranges and no adverse events reported. These findings support the feasibility of their use in this population under appropriate supervision.

Shorter-duration tests such as the 2MSPT and STST offer practical advantages, reducing evaluation time without compromising diagnostic value. This facilitates their implementation in adapted sports environments with logistical constraints, while also promoting more inclusive, accessible, and individualized assessments.

Finally, administering functional tests to individuals with intellectual disability requires methodological adaptations and increased attention to participant understanding and motivation. When these conditions are met, the application of these tests is both safe and effective, contributing to ongoing monitoring, tailored training, and the promotion of long-term healthy habits.

## Figures and Tables

**Table 1:** Descriptive data measurements for anthropometric variables, age and BMI.

	Mean ± SD	(95% CI) Min–Max
Age (years)	27.1 ± 5.6	(25.6 to 28.7) 18–35
Weight (kg)	72.7 ± 16.6	(68.1 to 77.4) 44.6–109.4
Height (cm)	173.9 ± 8.9	(171.4 to 176.4) 151.5–190.5
BMI	23.9 ± 4.6	(22.6 to 25.2) 17.0–34.0

Abbreviations: SD, standard deviation; CI, confidence interval.

**Table 2:** Descriptive performance in different tests.

TEST	Mean ± SD	(95% CI) Min–Max
6MWT (meters)	658.8 ± 76.5	(637.5 to 680.1) 473.0–802.0
STST (repetitions)	24.1 ± 4.4	(22.8 to 25.3) 14.0–32.0
2MSPT (steps)	90.7 ± 17.0	(88.4 to 96.5) 62.0–115.0
CST (steps)	158.8 ± 74.7	(138.0 to 179.7) 30–250

Abbreviations: SD, standard deviation; CI, confidence interval; 6MWT, Six-Minute Walking Test; STST, Sit-to-Stand Test; CST, Chester Step Test; 2MSPT, Two-Minute Step in Place Test.

**Table 3:** Comparison between the basal physiological response to exercise between different tests.

		STST	CST	2MSPT	6MWT	*p*–Value	*ηp* ^2^
SATURATION Basal	Mean ± SD	96.5 ± 1.4	94.5 ± 1.3	95.5 ± 1.6	95.5 ± 1.4		0.013
	(95% CI)	(96.0–96.9)	(93.0–98.9)	(94.0–97.9)	(94.03–97.9)	0.654	
	(min–max)	(93.0–100.0)	(92.0–99.0)	(92.5–100.0)	(89.0–98.0)		
HEART RATE Basal	Mean ± SD	68.9 ± 13.4	67.5 ± 11.9	67.0 ± 14.8	73.6 ± 13.7		0.047
	(95% CI)	(65.1–72.6)	(64.4–71.8)	(66.2–73.8)	(69.3–87.9)	0.112	
	(min–max)	(44–100)	(49–105)	(50–114)	(48–108)		
SYSTOLIC BLOOD TENSION Basal	Mean ± SD	116.5 ± 11.1	114.5 ± 9.1	113.6 ± 12.5	114.8 ± 3.3		0.027
	(95% CI)	(113.4–119.6)	(111.4–120.6)	(113.8–121.4)	(112.8–120.9)	0.342	
	(min–max)	(93.0–140.0)	(91.0–141.0)	(91.0–140.7)	(91.0–139.7)		
DIASTOLIC BLOOD TENSION Basal	Mean ± SD	73.4 ± 6.3	71.4 ± 6.9	78.3 ± 9.4	75.4 ± 17.7		0.016
	(95% CI)	(71.6–75.2)	(69.6–77.2)	(71.1–75.8)	(71.7–77.9)	0.567	
	(min–max)	(66.0–90.0)	(65.0–92.0)	(64–90.0)	(67.6–89.5)		

Abbreviations: SD, standard deviation; CI, confidence interval; 6MWT, Six-Minute Walking Test; 2MSPT, Two-Minute Step in Place Test; CST, Chester Step Test; STST, Sit-to-Stand Test. No post hoc pairwise comparisons were conducted for Table 2 because omnibus tests were non-significant for all baseline variables.

**Table 4:** Comparison between the final physiological response to exercise between different tests.

		STST	CST	2MSPT	6MWT	*p*-Value	*ηp* ^2^
SATURATION Final	Mean ± SD	95.6 ± 1.9	96.4 ± 1.7 *	96.1 ± 0.9	95.3 ± 1.5	0.006	0.096
	(95% CI)	(95.99.4)	(96.1–96.7)	(95.8–96.4)	(94.8–95.7)		
	(min–max)	(89–98)	(94–98)	(94–98)	(92–98)		
HEART RATE Final	Mean ± SD	82.7 ± 17.6	153.4 ± 21.3 *	85.0 ± 19.1	104.3 ± 20.7	<0.001	0.123
	(95% CI)	(77.8–87.6)	(147.2–159.9)	(79.2–90.9)	(98.5–110.1)		
	(min–max)	(54–122)	(110–201)	(60–136)	(62–148)		
SYSTOLIC BLOOD TENSION Final	Mean ± SD	123.6 ± 13.4	137.6 ± 18.1 *	129.0 ± 12.2	129.8 ± 12.7	<0.001	0.114
	(95% CI)	(119.9–127.3)	(132.2–143.0)	(125.3–132.7)	(126.2–133.3)		
	(min–max)	(100.0–151.0)	(102.1–169.5)	(115.0–156.4)	(109.5–157.2)		
DIASTOLIC BLOOD TENSION Final	Mean ± SD	78.8 ± 6.8	81.6 ± 7.7 *	77.6 ± 6.2	78.7 ± 5.8	0.018	0.078
	(95% CI)	(76.9–80.7)	(79.4–83.8)	(75.7–79.5)	(77.1–80.3)		
	(min–max)	(61.0–90.0)	(69.0–98.0)	(67.0–96.0)	(66.0–88.0)		

Abbreviations: SD, standard deviation; CI, confidence interval; 6MWT, Six-Minute Walking Test; 2MSPT, Two-Minute Step in Place Test; CST, Chester Step Test; STST, Sit-to-Stand Test. * Significantly different compared with the other tests according to post hoc pairwise comparisons conducted for Table 3.

**Table 5:** Correlation analysis between tests.

Comparison	r	95% CI	*p*-Value	FDR-Adjusted *p*
6MWT vs. STST	0.711	0.519–0.835	<0.001	<0.001
6MWT vs. CST	0.724	0.539–0.843	<0.001	<0.001
6MWT vs. 2MSPT	0.683	0.478–0.817	<0.001	<0.001

Abbreviations: 6MWT, Six-Minute Walking Test; 2MSPT, Two-Minute Step in Place Test; CST, Chester Step Test; STST, Sit-to-Stand Test; FDR, false discovery rate.

**Table 6:** Multivariable linear regression models predicting 6MWT distance.

Model (Predictor)	β (Unstandardized)	95% CI	*p*-Value	Standardized β	Adjusted R^2^
Model 1 (STST)	7.673	3.446–11.901	<0.001	0.448	0.264
Model 2 (CST)	0.076	0.011–0.141	0.024	0.305	0.161
Model 3 (2MSPT)	2.074	0.696–3.452	0.004	0.407	0.216

Abbreviations: 2MSPT, Two-Minute Step in Place Test; CST, Chester Step Test; STST, Sit-to-Stand Test.

## Data Availability

The data supporting the findings of this study are not publicly available due to confidentiality and/or proprietary restrictions. However, they can be made available to the journal for peer-review purposes upon request, subject to the appropriate permissions.

## Abbreviations

The following abbreviations are used in this manuscript:

1min-STST	One-Minute Sit-to-Stand Test
2MST	Two-Minute Step Test in Place
6MWT	Six-Minute Walk Test
BMI	Body Mass Index
Bpm	Beats Per Minute
CI	Confidence Interval
CST	Chester Step Test
DBP	Diastolic Blood Pressure
HR	Heart Rate
HRmax	Maximal Heart Rate
SBP	Systolic Blood Pressure
SD	Standard Deviation
SpO_2_	Oxygen Saturation
STROBE	Strengthening the Reporting of Observational Studies in Epidemiology
STST	Sit-to-Stand test
VO_2_max	Maximal Oxygen Uptake
WHO	World Health Organization
